# Vineyard Floor Management as a Multifunctional Strategy for Pollinator Communities and Grape Quality

**DOI:** 10.3390/plants15213405

**Published:** 2026-11-02

**Authors:** Eleonora Nistor, Alin Dobrei, Alina Andreea Dragoescu-Petrica, Eleonora Cataldo, Gabriel Ciorica, Alina Georgeta Dobrei

**Affiliations:** 1 Department of Horticulture, Faculty of Horticulture, Forestry and Plant Biotechnologies, University of Life Sciences “King Mihai I” from Timisoara, 119 Calea Aradului, 300645 Timisoara, Romania; nisnoranisnora@gmail.com (E.N.); alin1969tmro@yahoo.com (A.D.); 2 Department of Agricultural Technologies, Faculty of Agriculture, University of Life Sciences “King Mihai I” from Timisoara, 119 Calea Aradului, 300645 Timisoara, Romania; andreeadragoescu@usvt.ro; 3 Department of Agriculture, Food, Environment and Forestry Sciences and Technologies (DAGRI-UniFi), Florence University, 50100 Florence, Italy; eleonora.cataldo@unifi.it; 4 Department 6 Cardiology, Internal Medicine Ambulatory Discipline, “Victor Babes” University of Medicine and Pharmacy, Piata Eftimie Murgu 2, 300041 Timisoara, Romania

**Keywords:** bare soil, biodiversity, cover crops, grape quality, pollinators, spontaneous vegetation, vineyard floor management

## Abstract

In sustainable viticulture, inter-row vegetation management may improve biodiversity and influence grape quality, but the relationships among vegetation, pollinators, and grape quality remain insufficiently researched. A three-year study (2023–2025) was conducted in a vineyard in western Romania to evaluate the effects of inter-row management practices, including bare soil (BS), spontaneous vegetation (SV) and a multispecies cover crop mixture (CC), on vegetation traits, pollinator groups, grape yield and quality in four cultivars (‘Merlot’, ‘Pinot Noir’, ‘Sauvignon Blanc’ and ‘Italian Riesling’). Vegetation cover, flower density and plant species richness were recorded; pollinators were sampled using transects and classified into five groups; grape quality parameters, phenolic compounds, and anthocyanins (red cultivars) were measured at harvest time. CC consistently had the highest vegetation cover, flower density and species richness, whereas BS had the lowest values. Plant Shannon diversity was strongly positively associated with total pollinator abundance (r = 0.962, R^2^ = 0.925, *p* < 0.001; *n* = 108 plot-year observations), indicating that it accounted for 92.5% of the observed variation. Inter-row management explained 76.5% of the variation in pollinator community composition (PERMANOVA, R^2^ = 0.765, *p* = 0.001). Mean grape yield and berry weight were higher under BS (3.14 kg vine^−1^ and 1.45 g, respectively) than under SV (2.69 kg vine^−1^ and 1.36 g) or CC (2.29 kg vine^−1^ and 1.29 g; *p* < 0.001). Total phenolic content was higher under CC than under BS in all four cultivars, although the magnitude of the difference depended on cultivar. In the two red cultivars, mean anthocyanin content was higher under CC (751 mg L^−1^) than under SV (672 mg L^−1^) or BS (621 mg L^−1^). Treatment differences in vegetation characteristics varied among years, while grape quality responses also differed among cultivars. Overall, the results indicate a trade-off between yield-related traits, which were associated with BS, and phenolic and anthocyanin accumulation, which were higher under CC. Multispecies cover crops may therefore offer ecological and compositional benefits, although their use should account for potential competition with grapevines for resources.

## 1. Introduction

The decline of pollinating insect communities is a challenge to the implementation of sustainable agriculture and conservation of global biodiversity [1,2]. Reductions in population abundance and species diversity among groups, such as honeybees and bumblebees, butterflies, and syrphid flies, have been widely observed over the past several decades, especially in agricultural environments [3]. Intensive agricultural practices, such as monocultures, heavy soil tillage, and the widespread and frequent application of pesticides, can negatively affect plant and pollinator diversity and their interactions, thereby impairing ecosystem functioning and stability [4,5]. Unlike many entomophilous crops, grapevine (*Vitis vinifera*) is a plant with predominantly hermaphroditic flowers, and most wine grape cultivars are self-pollinating and wind pollinated [6], requiring no insect vectors for fruit set. Pollinators provide an indispensable service in terrestrial ecosystems by enhancing crop production and the resilience of plant communities [7,8]. Impacts on crop production, biological regulation, and the stability of ecological systems have been associated with losses in pollinator population and diversity [9]. Hence, preserving plant diversity and pollinator populations and increasing habitat availability are key priorities for sustainable agricultural management [10]. In agriculturally dominated landscapes, plant resource abundance and temporal stability are considered key predictors of pollinator presence and community structure [11]. The extent of these responses depends on local vegetation composition and land management, which can vary greatly between agroecosystems [10,12].

Increasing landscape diversity and implementing agri-environmental strategies can enhance insect–plant interactions and promote pollinator diversity in agricultural ecosystems [10,11]. During the flowering period, plant community diversity provides pollinators with shelter and nesting sites while also providing nectar and pollen resources [12]. Vegetation community composition can enhance functional diversity by increasing floral abundance [13]. Functional biodiversity refers to the diversity of species that support important ecosystem services, such as pollination, biocontrol, and nutrient cycling [14]. Therefore, maintaining and promoting diverse cover crops has become increasingly important in sustainable development strategies aimed at conserving biodiversity and enhancing ecosystem function [15]. However, pollinator responses may not be fully explained by plant species richness alone, as the abundance, richness and seasonal timing of flowering also influence resource availability for different insect guilds [11]. Examining vegetation metrics along with pollinator abundance can thus shed light on ecological associations that follow different management options [14].

Vineyards are established for the production of grapes for wine or direct consumption, but they are also permanent agroecosystems that can support appreciable biodiversity when managed appropriately [16]. Inter-row vegetation and semi-natural habitats surrounding vineyards can act as ecological infrastructures that provide both trophic resources and habitat for pollinators, predatory insects, birds and soil organisms [17]. Grapevine is a plant with predominantly hermaphroditic flowers, and most wine grape cultivars are self-pollinating [18]. Thus, the contribution of insect pollinators to grapevine fruit set is unlikely to be comparable to their contribution to crops that rely on animal-mediated pollination [19]. In vineyard systems, the ecological importance of pollination services can also be interpreted in terms of biodiversity conservation and the perpetuation of relevant ecosystem functioning [20]. Vegetation may also provide habitat and resources for natural enemies of vineyard pests; however, the presence of vegetation and pollinators alone does not demonstrate effective biological pest control [17]. These aforementioned benefits may serve as an ecological rationale for vineyard vegetation management but are not a substitute for direct evaluation when these effects are not explicitly measured.

Among the management practices available to vineyard producers, managing vegetation between rows is the practice most applicable since it controls the development and makeup of the vegetated area in the inter-row area [14]. The ecological value of vineyards is influenced by inter-row vegetation management within vine rows and inter-rows [21]. These strategies also influence vegetation, insect diversity and community structure [22]. Conventional bare soil inter-row management or herbicide treatments minimise competition for nutrients between vines and spontaneous vegetation but also decrease biodiversity and increase exposure to erosion [23]. In recent decades, in many vineyards, spontaneous vegetation or sown cover crops have increasingly been practised in the inter-row to improve soil quality by incorporating organic matter, to increase the above- and below-ground faunal biodiversity, or to reduce erosion [24,25,26]. Cover crops may additionally provide a more predictable and manageable source of floral resources, whereas spontaneous vegetation can support naturally occurring and potentially heterogeneous plant communities [27]. These contrasting management practices could potentially influence the species composition of pollinator communities through changes in vegetative cover, plant species diversity and floral resource availability [28]. Bare soil is expected to provide less of a resource than vegetated inter-rows, although sown and spontaneously developed vegetation provides contrasting patterns of floral resources due to differences in species richness, flowering morphology, and timing [29]. Greater vegetation and floral resources could thus facilitate increased pollinator activity and abundance, although the strength of any such relationship could vary between insect groups, seasons, and vineyard sites. A more complex effect, however, is associated with vineyard vegetative cover in relation to grape production. Vegetation might contribute to increased protection of the soil and stimulate activity of soil biota but could also compete with grapevines for water and nutrients, especially in the absence of irrigation [25]. As such, the magnitude and direction of the relationships between vineyard productivity and composition and the extent of inter-row vegetation cannot be known a priori. Thus, simultaneous examination of vineyard floristic, pollinator communities, and grape growing is essential to ensure that the ecological responses (pollinating bee activity) associated with the presence or absence of inter-row vegetation are consistent with measurable vineyard responses [29].

According to recent studies, inter-row vegetation improves biodiversity and ecosystem services in vineyards, but much of the available evidence is from France, Spain, or Italy [28,29,30]. Few studies have simultaneously investigated how different inter-row vegetation management strategies affect vegetation characteristics, pollinator populations, and grape compositional attributes throughout several growing seasons in an Eastern European vineyard [31,32]. Integrated assessments of bare soil inter-row management, sown cover crops, and spontaneous vegetation in multiple years and for red and white wine grape cultivars can help address the lack of evidence within the region concerning the association between the above-mentioned management options and ecological and production-related responses [22,29]. The particular research lacuna tackled here is the dearth of information on the link between the traits of inter-row vegetation, pollinator abundance, grape yield, and grape composition under the environmental conditions of western Romania [22]. Explaining this gap first requires separating the comparison of vegetation management treatments from correlations among the measured variables: while the former can help reveal the differences among management options, the latter can determine associations but not causal mechanisms [29].

Therefore, the purpose of this study was to evaluate the relationships between contrasting inter-row vegetation management practices and vegetation characteristics, pollinator abundance and community composition, grape yield, and grape compositional quality in a vineyard in western Romania. It also examined the associations between vegetation attributes and pollinator abundance and compared grape production and quality parameters among the three management treatments and four cultivars. The experiment consisted of three inter-row vegetation treatments (bare soil—BS, spontaneous vegetation—SV, multispecies cover crop mixture—CC) and assessed the vegetation cover and flower density, species richness, flower and plant community composition; pollinator abundance and community composition; and vineyard yield and composition quality.

Specifically, the primary goals were to: (i) compare the three inter-row vegetation management treatments in terms of vegetation cover, floral resources, and plant species richness; (ii) assess differences in the abundance and community composition of bees, bumblebees, hoverflies, and butterflies among treatments; (iii) evaluate the relationships between vegetation attributes and pollinator abundance without assuming causal effects; and (iv) compare grape yield and compositional quality parameters among treatments and cultivars.

We hypothesised that inter-row vegetation management would be associated with differences in vegetation characteristics and pollinator communities, and that vegetation attributes would be associated with pollinator abundance; we also expected grape yield and compositional quality responses to vary among management treatments and cultivars (‘Merlot’, ‘Pinot Noir’, ‘Sauvignon Blanc’ and ‘Italian Riesling’).

## 2. Results

### 2.1. Effects of Inter-Row Management on Vineyard Floor Vegetation

Inter-row management and year significantly influenced vegetation cover, flower density, and plant species richness, with significant Treatment × Year interactions indicating that treatment effects varied across years (Table 1).

**Figure 1:**
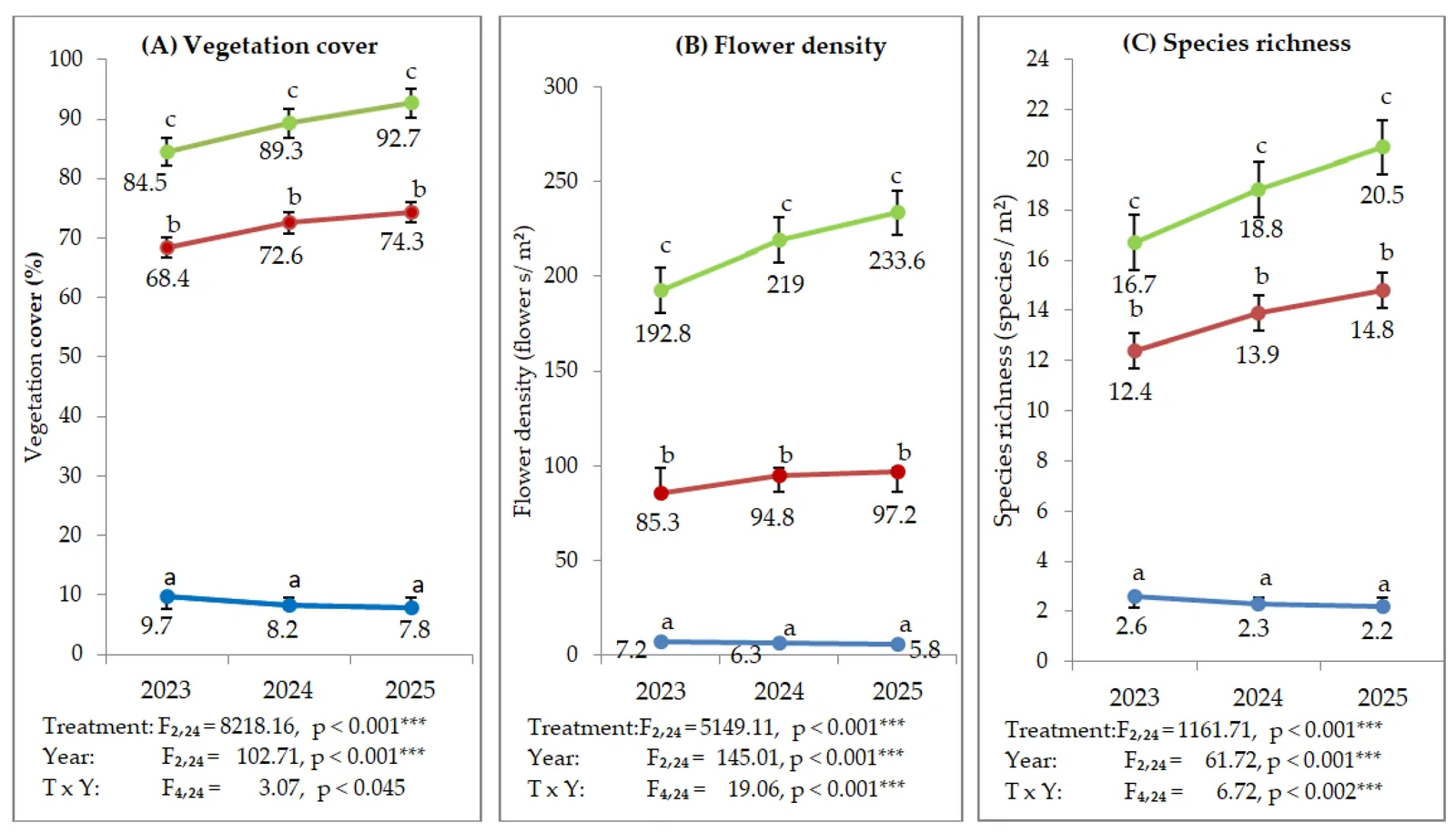
Distribution of vegetation cover (**A**), density of flowers (**B**), and species richness (**C**) in relation to inter-row management treatments (cover crop mixture = CC; spontaneous vegetation = SV; bare soil = BS) over three successive vegetation periods (2023–2025) in a commercial vineyard located in the Minis-Maderat vineyard region, western Romania. Data are average ± SD (*n* = 3 plots per treatment). Different lowercase letters within each year denote significant differences between treatments, as revealed by Tukey’s HSD test (*p* < 0.05). Results from the two-way ANOVA for Treatment (T), Year (Y), and their interaction (T × Y) are presented at the bottom of each panel, with asterisks indicating statistical significance (*** *p* < 0.001).

Vegetation cover expanded throughout the research duration under both cover crop (CC) and spontaneous vegetation (SV) management, while bare soil (BS) had persistently low vegetation cover. Flower density followed a similar pattern, with the highest annual mean under CC, intermediate values under SV, and significantly lower values for BS throughout the study period. Plant species richness followed a similar treatment gradient, with CC maintaining the most diverse plant community, followed by SV, and BS consistently supporting the fewest species. CC consistently had the highest values for all three vegetation traits, followed by SV and BS. Post hoc comparisons indicated significant differences among treatments (Tukey’s HSD, *p* < 0.05; Figure 1).

Year-specific treatment means (±SE) and Tukey-adjusted pairwise comparisons for vegetation cover, flower density, and Shannon diversity (H′) are presented in Supplementary Table S1; species richness is shown separately in Figure 1C.

### 2.2. Pollinator Community Composition

**Figure 2:**
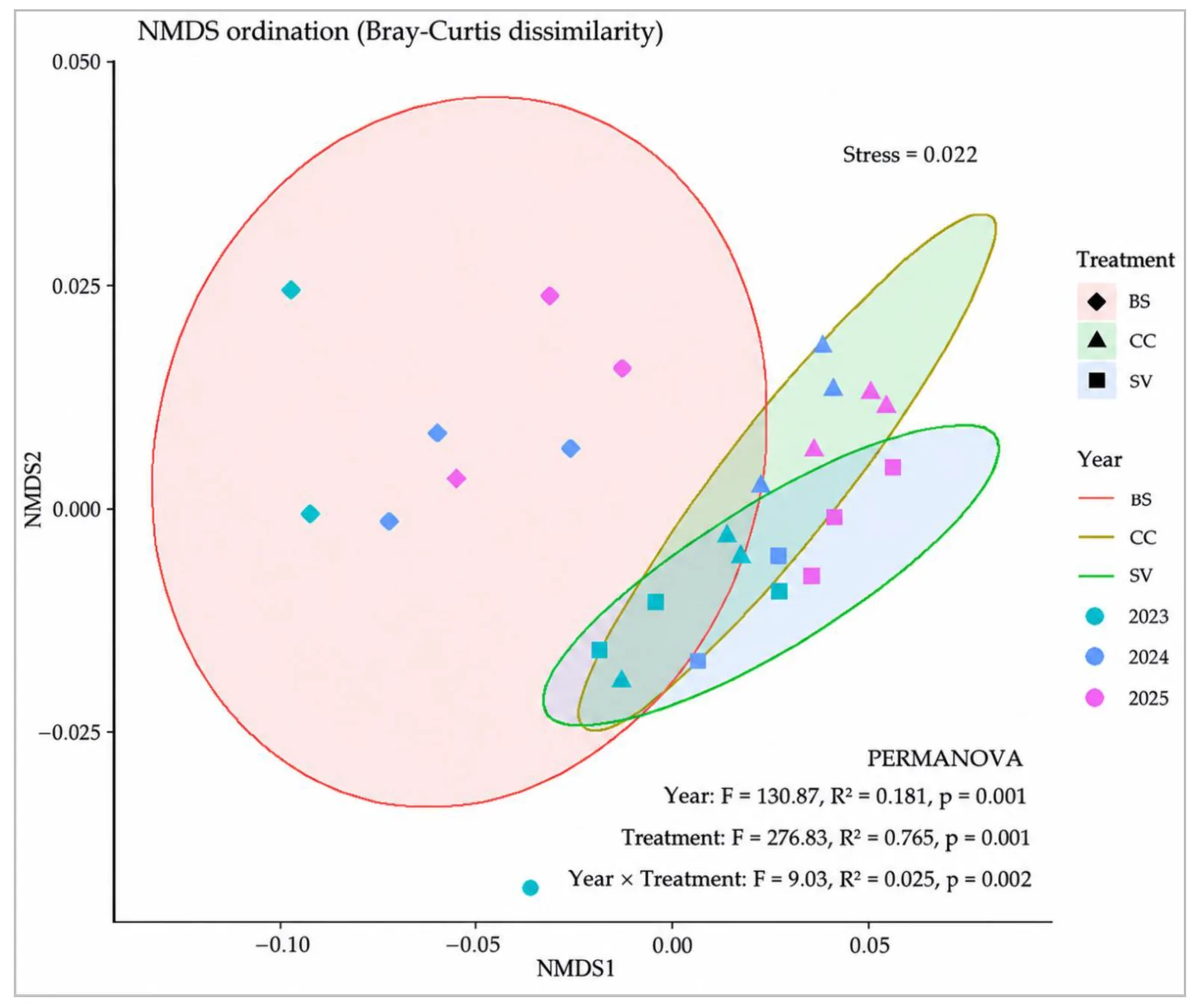
Non-metric multidimensional scaling (NMDS) ordination of pollinator communities across different inter-row management treatments—bare soil (BS), spontaneous vegetation (SV), and cover crop mixture (CC)—evaluated in a commercial vineyard in western Romania during the 2023–2025 seasons. Ellipses represent 95% confidence intervals. PERMANOVA test results (F-statistic, R^2^, and *p*-values) for the main effects of treatment, year, and their interaction (Treatment × Year) are displayed in the lower right corner.

The NMDS ordination based on Bray–Curtis dissimilarities provided a very good representation of pollinator community structure (stress = 0.022), revealing distinct patterns across the three inter-row management systems (Figure 2). Samples from the BS treatment were distributed more widely in the ordination space, whereas CC and SV samples exhibited partially overlapping clusters with overlapping 95% confidence ellipses, indicating a higher degree of similarity in pollinator community composition within the two vegetation-based treatments.

A two-factor PERMANOVA based on Bray–Curtis dissimilarities revealed significant effects of inter-row management treatment, sampling year, and their interaction on pollinator community composition (Table 2). The largest proportion of variation in community composition was explained by inter-row management (R^2^ = 0.765, *p* = 0.001), followed by sampling year (R^2^ = 0.181, *p* = 0.001). The Treatment × Year interaction explained a smaller proportion of variation (R^2^ = 0.025) but was still significant (*p* ≤ 0.002). The analysis was based on *n* = 108 plot-year observations (36 plots × 3 years) with 999 permutations. No significant differences in multivariate dispersion were identified by PERMDISP between treatments (*p* = 0.868) or years (*p* = 0.982), indicating that the significant PERMANOVA results were related to differences in community composition rather than differences in within-group dispersion.

### 2.3. Responses of Pollinator Groups to Inter-Row Vegetation Management

**Figure 3:**
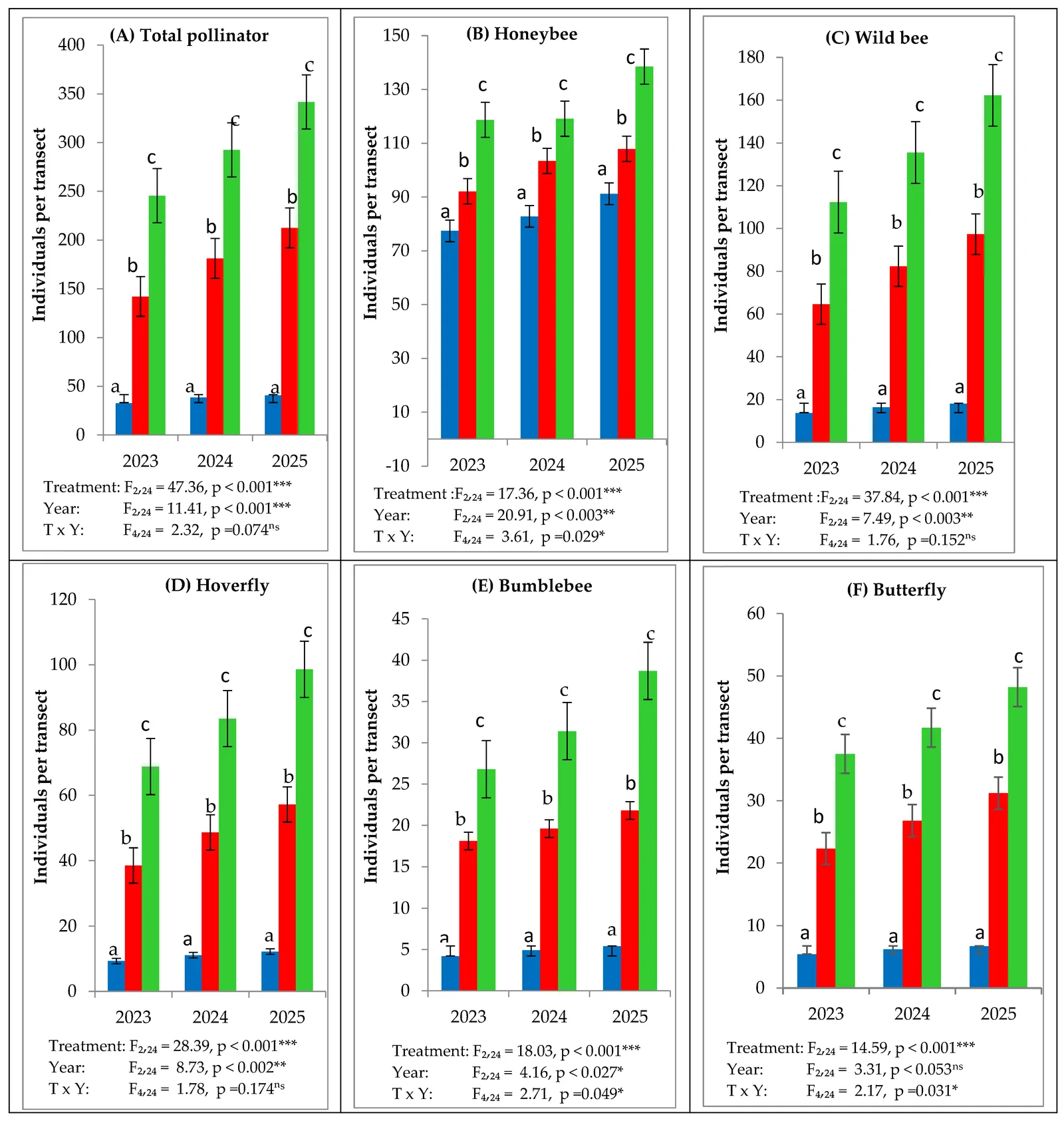
Total pollinator abundance and responses of main pollinator functional groups (honeybees, wild bees, hoverflies, butterflies, and bumblebees) across different inter-row management treatments—cover crop mixture (CC), spontaneous vegetation (SV), and bare soil (BS)—evaluated across three consecutive growing seasons (2023–2025) in a commercial vineyard in western Romania. Values represent the mean ± SD (*n* = 3 plots per treatment). Different lowercase letters within each year indicate statistically significant differences among treatments according to Tukey’s HSD test (*p* < 0.05). Significance levels for Treatment, Year, and their interaction (T × Y) are indicated as follows: ns, not significant (*p* > 0.05); * *p* < 0.05; ** *p* < 0.01; *** *p* < 0.001.

The abundance of pollinators increased from 2023 to 2025 for all three inter-row management treatments, with the highest abundance consistently observed under CC and the lowest under BS. Honeybees were the most abundant pollinator group, and their mean numbers were always higher under CC than under SV and BS. Wild bees showed the strongest numerical response to vegetation-based management, although hoverflies, bumblebees, and butterflies were all substantially more abundant under CC and SV than under BS (Figure 3).

Year effects were significant for total pollinator abundance, honeybees, wild bees, hoverflies, and bumblebees. The Treatment × Year interaction was significant for honeybees and bumblebees, indicating that the magnitude of treatment differences varied across years. Total pollinator abundance, wild bee abundance, and hoverfly abundance showed no significant Treatment × Year interaction, indicating that the treatment patterns for these groups were comparatively consistent across the study period. For butterflies, the Treatment × Year interaction was marginal (*p* = 0.053) and therefore did not reach the conventional significance threshold of *p* < 0.05 (Figure 3).

Year-specific treatment means (±SE) and Tukey-adjusted pairwise comparisons for each pollinator group are provided in Supplementary Table S2.

### 2.4. Relationships Between Vegetation Characteristics and Pollinator Abundance

**Figure 4:**
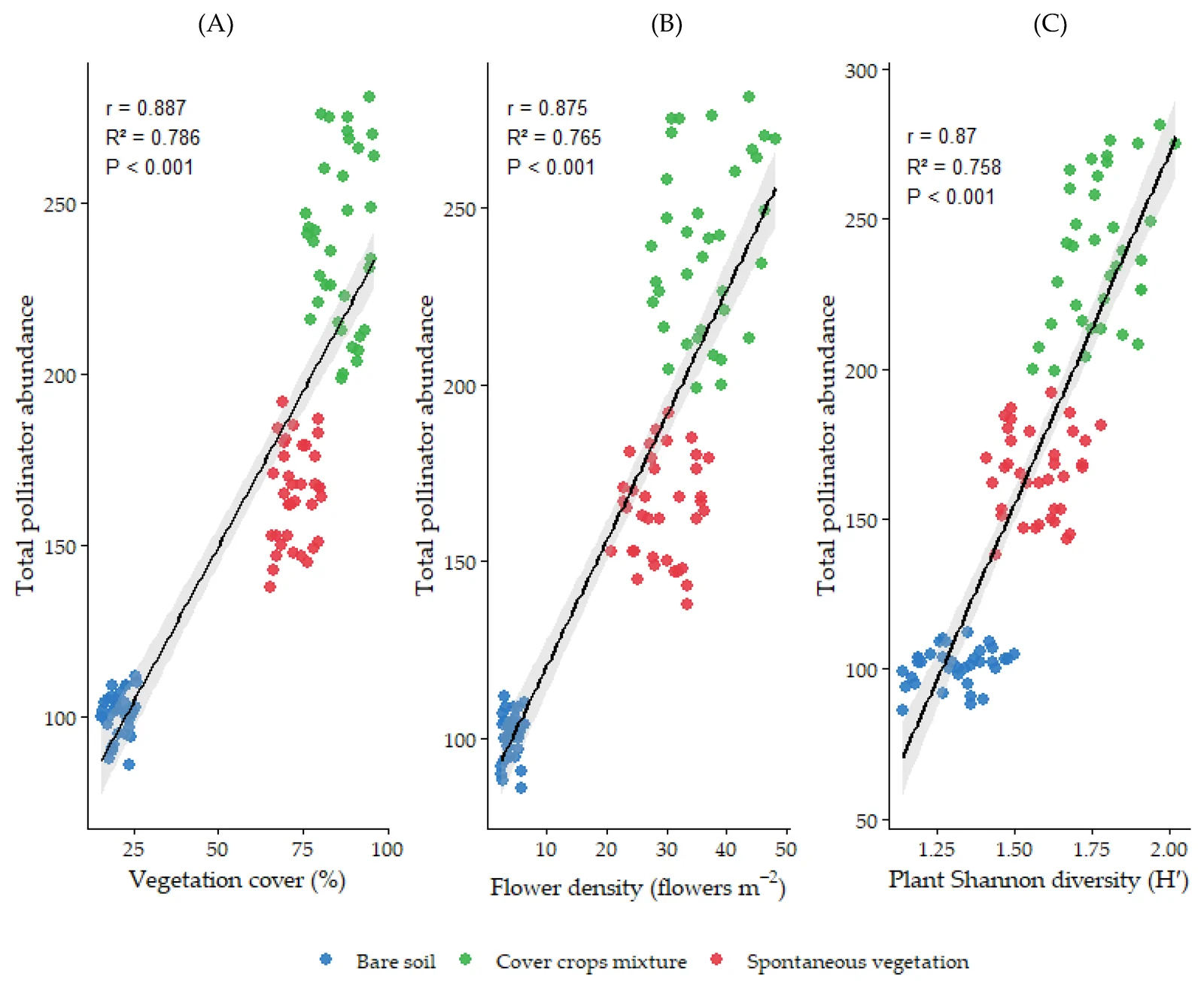
Relationships between vineyard floor vegetation characteristics and total pollinator abundance across different inter-row management treatments—cover crop mixture (CC), spontaneous vegetation (SV), and bare soil (BS)—evaluated during the 2023–2025 seasons in a commercial vineyard in western Romania. Linear regressions show the relationships between total pollinator abundance and (**A**) vegetation cover (%), (**B**) flower density (flowers∙m^−2^), and (**C**) plant Shannon diversity (H′). Points represent individual sampling plots (*n* = 36) color-coded by inter-row management treatment. Solid lines indicate fitted linear regression models, and shaded areas represent 95% confidence intervals.

Total pollinator abundance was significantly positively related to all three explanatory variables: vegetation cover, flower density, and plant Shannon diversity across the 108 plot-year observations (Figure 4). Linear regression analyses showed positive relationships between total pollinator abundance and vegetation cover (Figure 4A; r = 0.778, R^2^ = 0.606, *p* < 0.001) and flower density (Figure 4B; r = 0.766, R^2^ = 0.587, *p* < 0.001). Plant Shannon diversity showed the strongest single bivariate relationship with pollinator abundance (Figure 4C), with the reported model explaining approximately 92.5% of the variation. Furthermore, a multiple linear regression model including flower density and Shannon diversity explained 83.4% of the total variation in the abundance of pollinators (Table 3).

Pollinator abundance and vegetation characteristics also differed among inter-row vegetation management treatments (Figure 4). Bare soil (BS) plots had the lowest vegetation cover, flower density, plant Shannon diversity, and pollinator abundance. Spontaneous vegetation (SV) and cover crop mixture (CC) plots generally had higher values for these vegetation characteristics and higher pollinator abundance, with the highest values generally observed under CC.

Combined, these findings show that pollinator abundance in the vineyard floor was positively associated with both floral resource abundance and local plant species richness, and that only management regimes that foster production of more flowers in more diverse plant communities (including spontaneous vegetation and, particularly, cover crop mixtures) were associated with increased pollinator abundance.

### 2.5. Multivariate Relationships Between Inter-Row Vegetation Management and Grape Quality and Yield

**Figure 5:**
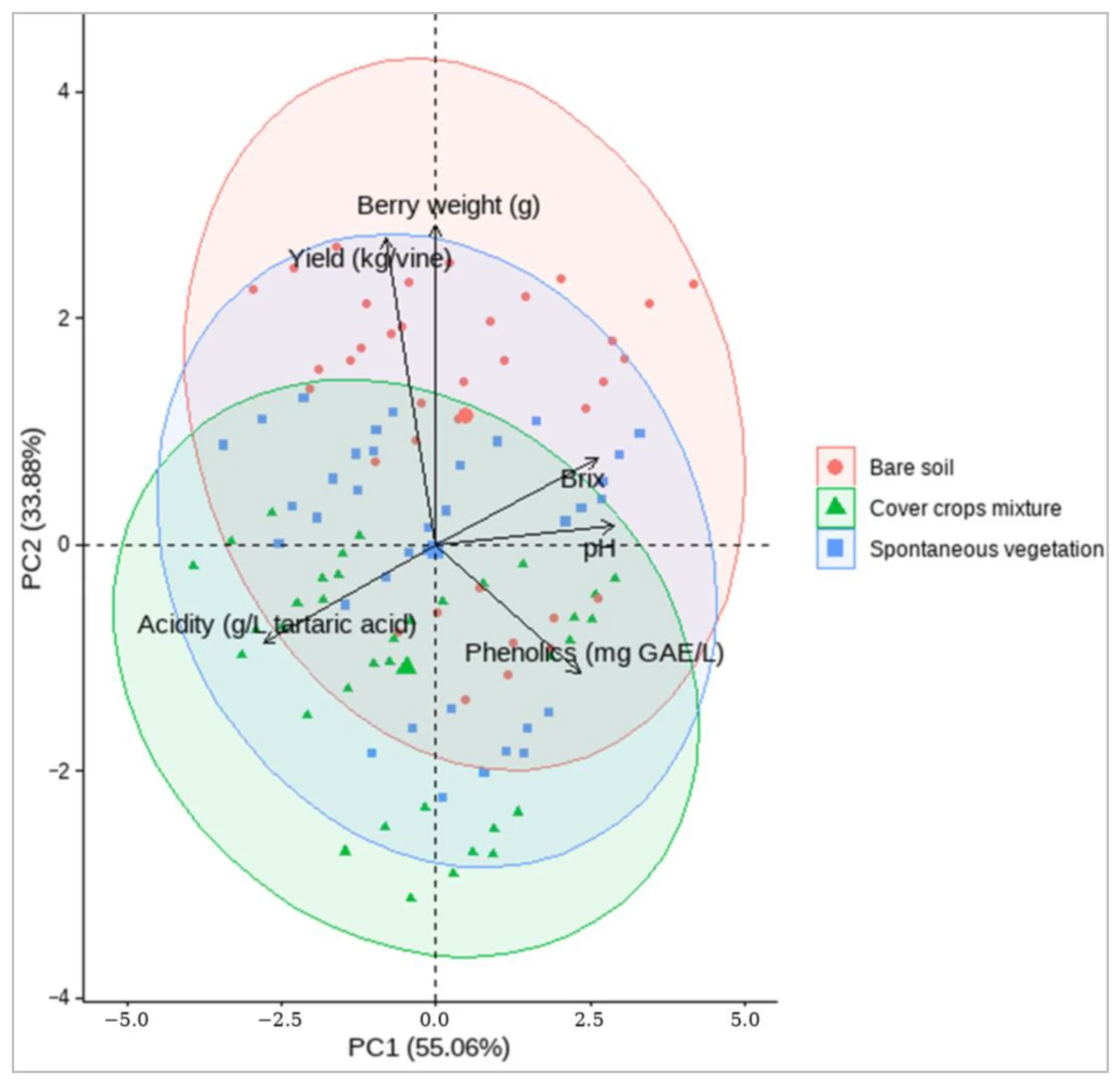
Principal component analysis (PCA) biplot of grape yield and quality parameters for the white grape cultivars (‘Sauvignon Blanc’ and ‘Italian Riesling’) evaluated under different inter-row management treatments—bare soil (BS), spontaneous vegetation (SV), and cover crop mixture (CC)—over the 2023–2025 seasons in a commercial vineyard in western Romania. Arrows represent variable loadings, and ellipses indicate 95% confidence intervals around treatment centroids.

Principal component analysis (PCA) was performed to analyse multivariate relationships between soil inter-row vegetation management treatments and grape quality and yield parameters in two white cultivars (‘Sauvignon Blanc’ and ‘Italian Riesling’). The first two principal components explained most of the total variation, with PC1 and PC2 explaining almost 88.94% of the variance (PC1 = 55.06%; PC2 = 33.88%; Figure 5). PC1 primarily represented a gradient in grape composition, with positive loadings for pH, soluble solids (°Brix), and phenolic content and a negative loading for titratable acidity. PC2 reflected variation associated with yield-related traits, including yield and berry weight.

Grape compositional characteristics were clearly distinguished by the first principal component (PC1). Samples with higher values on this axis were characterised by increased pH, soluble solids (°Brix) and total phenolic content, while titratable acidity was strongly negatively associated with PC1.

This component therefore represents a gradient of grape ripeness and compositional quality, with higher PC1 values suggesting advanced ripening and higher phenolic composition. By comparison, the second principal component (PC2) was mostly affected by physical yield-related traits (berry weight and total yield), distinguishing treatments based on productivity instead of chemical composition. The biplot showed different clustering patterns of inter-row vegetation management practices. Samples from bare soil (BS) were found to be situated on the positive side of PC2, in the direction of the berry weight and yield vectors, indicating a relationship with higher values of these parameters. Cover crop mixture (CC) samples had a stronger relationship with the compositional characteristics represented along PC1, notably phenolic content, whereas titratable acidity was oriented in the opposite direction. Samples from spontaneous vegetation (SV) were more widely scattered over the PCA space, indicating more variability in combined quality and yield attributes.

These multivariate patterns were complemented by direct comparisons of treatment means (Table 4). BS had higher yield (3.14 ± 0.03 versus 2.29 ± 0.03 kg/vine; Tukey-adjusted *p* < 0.001) and berry weight (1.45 ± 0.01 versus 1.29 ± 0.01 g; *p* < 0.001) than CC. The pooled mean phenolic content was higher under CC (1056 ± 6.8 mg GAE/L) than under SV (960 ± 6.8 mg GAE/L) and BS (893 ± 6.8 mg GAE/L). However, because the Treatment × Cultivar interaction was significant (F_6,72_ = 12.82, *p* < 0.001), these pooled means should be interpreted descriptively; cultivar-specific comparisons are presented in Table 5. CC had higher phenolic content than BS in all four cultivars, although the significance of pairwise comparisons involving SV varied among cultivars. Treatment × Cultivar interactions were non-significant for yield, berry weight, soluble solids (°Brix), pH, and titratable acidity (all *p* > 0.05; Table 4), indicating that treatment differences for these traits did not vary significantly among cultivars. Overall, the results associate BS with higher yield-related traits and CC with higher phenolic content, although the magnitude of the phenolic response differed among cultivars.

### 2.6. Multivariate Relationships of Inter-Row Management and Red Grape Quality

**Figure 6:**
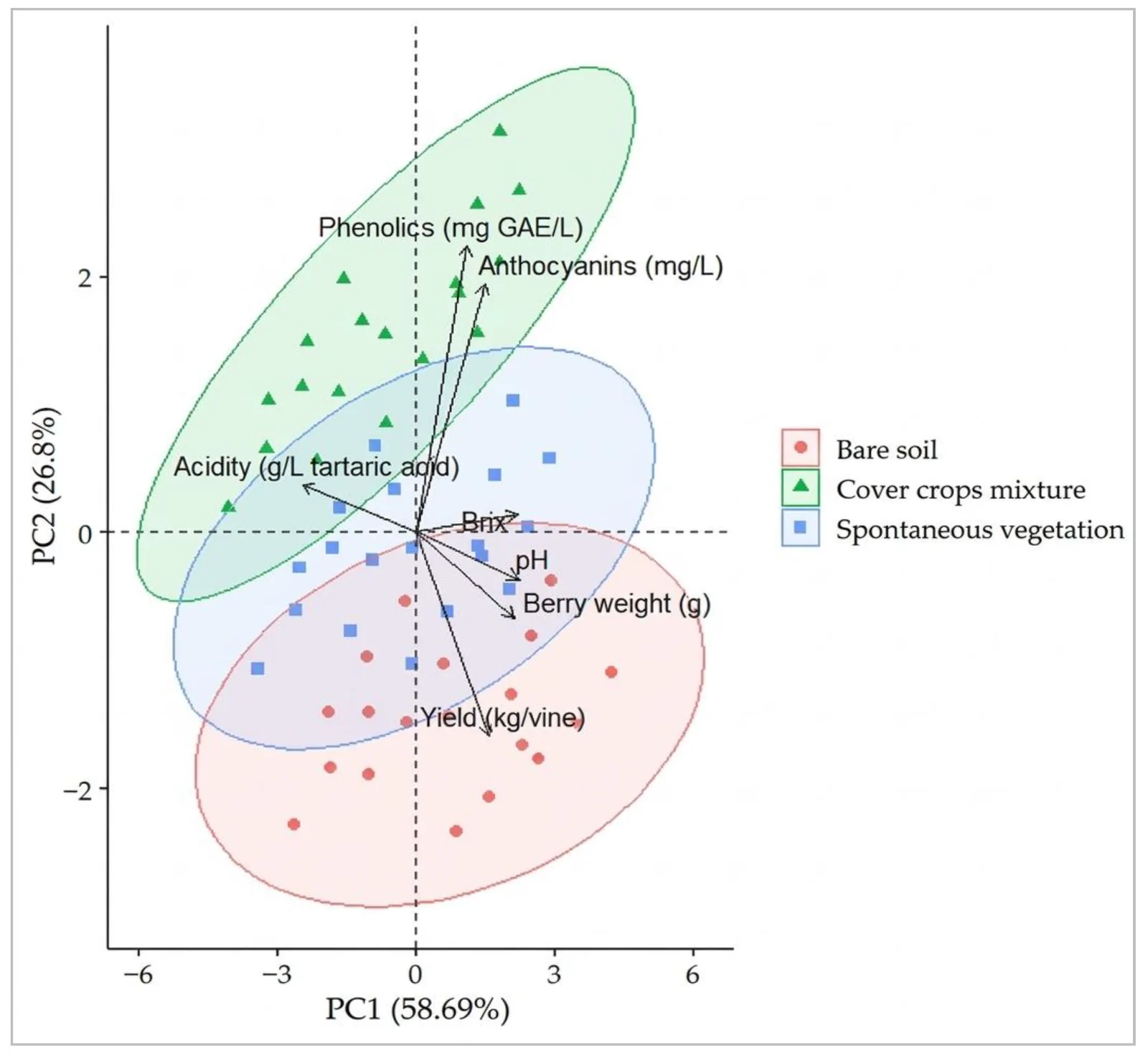
Principal component analysis (PCA) biplot showing the relationships between grape yield-related variables and quality attributes in the red grape cultivars (‘Merlot’ and ‘Pinot Noir’) evaluated across three inter-row management treatments—cover crop mixture (CC), spontaneous vegetation (SV), and bare soil (BS)—over three consecutive growing seasons (2023–2025) in a commercial vineyard in western Romania. Arrows represent variable loadings, whereas ellipses denote 95% confidence intervals around treatment centroids.

Principal component analysis (PCA) was used to examine the multivariate relationships between grape quality and yield traits for the red cultivars (‘Merlot’ and ‘Pinot Noir’). More than 85% of the variance was explained by the first two principal components, PC1 and PC2 (Figure 6). Titratable acidity showed a negative loading on the first principal component (PC1), whereas pH and soluble solids (°Brix) showed positive loadings; PC1 therefore primarily reflected a gradient in ripening-related compositional characteristics. The second principal component (PC2) was associated with productivity-related traits and secondary metabolite accumulation.

The sample distribution in the PCA biplot (Figure 6) revealed variations across the inter-row vegetation management strategies, although the 95% confidence ellipses showed some overlap. Samples from bare soil (BS) were more likely to occur in the direction of the berry weight and yield vectors, showing a relationship with higher values of productivity-related traits. In contrast, samples from cover crop mixtures (CC) were situated closer to phenolic compounds, anthocyanins, and titratable acidity, indicating improved compositional quality.

Direct comparisons of treatment means (Table 4 and Table 5) confirmed the multivariate patterns. The treatment with the highest yield (3.14 ± 0.03 vs. 2.29 ± 0.03 kg/vine; Tukey-adjusted *p* < 0.001) and berry weight (1.45 ± 0.01 vs. 1.29 ± 0.01 g; *p* < 0.001) was BS, while CC was the highest in phenolics (Table 5) and anthocyanins (751 ± 7.7 vs. 621 ± 7.7 mg/L; *p* < 0.001). SV treatment was intermediate for both trait groups.

Treatment × Cultivar was not significant for anthocyanins (*F*_2,36_ = 2.59, *p* = 0.089), indicating that the differences among treatment effects did not vary significantly between ‘Merlot’ and ‘Pinot Noir’ for this trait.

### 2.7. Explanatory Capacity of Models for Grape Quality and Yield

The complete factorial models explained a substantial proportion of the variation in grape quality and yield parameters (Table 6). The models explained 79.9–99.6% of the variance, with adjusted R^2^ ranging from 0.701 to 0.994. Phenolic compounds showed the highest explanatory capacity, whereas soluble solids (°Brix) showed the lowest, although the model still explained 79.9% of the observed variance. Diagnostic analyses showed no influential observations and no evidence of multicollinearity (VIF = 1.00). Some models showed deviations from residual normality, most notably for phenolic compounds. These diagnostic data were considered when interpreting the model estimates.

## 3. Discussion

### 3.1. Effects of Vegetation Management on Vineyard Floor Species and Pollinator Resources

The results of this study suggest that inter-row management practices significantly and consistently affected vegetation characteristics, with cover crop mixtures resulting in the highest vegetation cover, flower density and species richness in all three growing seasons. This is consistent with previous studies showing that sown cover crop mixtures increase the availability of floral resources and enhance plant diversity compared to spontaneous vegetation or bare soil inter-row management [33,34,35]. In a European survey of 63 vineyards, extensive vegetation management and enhanced floral resource availability were linked to increased wild bee abundance and diversity, whereas less intensive management resulted in much higher inter-row vegetation cover [36]. Similarly, studies comparing vegetated inter-rows have found positive relationships between plant diversity and arthropod abundance, indicating the function of plant communities as an essential component of vineyard habitat quality [37].

All vegetation metrics showed significant Treatment × Year interactions, implying that treatment effects were dynamic and became more pronounced throughout the study. The cover crop treatments exhibited incremental increases in vegetation cover from 84.5% in 2023 to 92.7% in 2025, suggesting that perennial or self-seeding combinations can develop deeper root systems and become more competitive over time. In contrast, bare soil inter-row treatments consistently had low values for all vegetation metrics, indicating that tillage or herbicide application suppressed spontaneous plant establishment. The observed treatment ranking—cover crop combination > spontaneous vegetation > bare soil—was consistent in terms of flower density and plant species richness, supporting the view that active vegetation management may significantly improve floral resources in vineyard settings.

These outcomes support recent research from Northern Italy, which found herbaceous vegetation cover and floral diversity as major factors positively affecting pollinator abundance and diversity [38]. Similarly, studies from southern France have revealed strong associations between Hymenoptera abundance, floristic richness, and plant cover, highlighting the consistency of these relationships across viticultural regions [39].

### 3.2. The Composition of Pollinator Communities Reflects the Availability of Resources Provided by Vegetation

The NMDS ordination showed clear differences in pollinator communities among treatments, with the bare soil samples forming a clear cluster compared with the partially overlapping communities under cover crop and spontaneous vegetation treatments. This pattern indicates that vegetated inter-row treatments support more similar pollinator communities, probably due to comparable plant resources. Inter-row management treatment was also the dominant factor structuring pollinator communities, explaining >76% of the variance in community composition, far beyond the contribution of year (18%) or the ‘Treatment × Year’ interaction (3%). The strong effect of local vegetation management is in agreement with other vineyard studies which showed that ground vegetation characteristics were major predictors of wild bee community structure and often more important than landscape context or local influences [36]. In this regard, substantial vegetation management and improved floral resource availability have been linked to enhanced wild bee abundance throughout vineyards in different European countries [40].

Responses of individual pollinator groups give further insight into the ecological mechanisms behind these community-level patterns. Cover crop and spontaneous vegetation treatments had higher numbers of honeybees, wild bees, bumblebees, and butterflies than bare soil; wild bees had the highest numerical response to vegetation-based management. This differential sensitivity may reflect the dependence of many wild bee species on a diversity of floral resources and adequate nesting sites, which are more abundant and varied in vegetated inter-rows. The uniform ranking of treatments over the years indicates that the benefits of vegetation management are temporally stable and such stability may have important implications for the adoption of pollinator-friendly practices in viticulture [41]. The stability of treatment differences over years indicates that the effect of inter-row vegetation management was rather steady across the three-year research period. This temporal stability is important for vineyard management because it shows that the ecological advantages of vegetated inter-rows extended beyond a single growing season.

### 3.3. Plant Diversity Is the Best Predictor of Pollinator Abundance

Both vegetation cover and flower density were positively associated with total pollinator abundance, but plant diversity, expressed as the Shannon index, showed the strongest relationship with pollinator abundance. Plant diversity alone explained more than 92% of the observed variation, while the multiple regression model retained plant diversity as the only significant predictor (β = 0.890, *p* < 0.0001). Flower density did not provide a significant additional contribution after plant diversity was included in the model (β = 0.094, *p* = 0.269). Thus, within the conditions of the present study, the diversity of the plant community was more closely associated with pollinator abundance than flower density considered independently.

This result is consistent with findings from vineyards and other agricultural systems that plant variety and floral resource diversity can be major predictors of pollinator populations. Kratschmer et al. [41] found a favourable relationship between the functional richness of blooming plants and the functional richness of wild bees in vineyard inter-rows, and they suggested varied cover crop mixes to increase flowering plant variety.

The recognition of plant diversity as the most significant characteristic of vegetation has practical consequences for vineyard management [29,42]. Results suggest that management should focus on establishing and sustaining the maintenance of diverse plant communities rather than maximising vegetation cover or floral abundance. This is in accordance with previous research recommendations to use site-adapted combinations of native wild plants with a variety of flower colours, shapes, and phenologies to sustain diverse pollinator groups [43]. The tight confidence intervals along the diversity gradient suggest a strong relationship between Shannon diversity and pollinator abundance, which contributes to the evidence that increasing plant diversity, is a reliable way to promote pollinator conservation in viticultural landscapes. The ecological interpretation of the current findings is that a more diversified plant community may supply a greater range of floral resources that differ in species identity, blooming season, and floral features. The regression analysis shows strong associations rather than causal effects; therefore, the high R^2^ should be understood within the study’s geographical and temporal context.

### 3.4. Differentiated Effects of Inter-Row Management on Grape Yield and Quality

Multivariate evaluations of grape quality parameters revealed that inter-row management strategies had distinct impacts on white and red grape cultivars, although overall trends remained quite similar across both cultivar groups. For white grape cultivars (‘Sauvignon Blanc’ and ‘Italian Riesling’), PC1 was a gradient of technological maturity (the optimal balance between sugar accumulation, titratable acidity, and must pH for winemaking) and chemical composition quality, with cover crop samples closer to titratable acidity and phenolic concentrations, and bare soil samples related to berry weight and yield. PC2 had a stronger correlation with berry weight and yield, showing a distinction between compositional and productivity-related features. Within this multivariate space, bare soil samples were more closely related to berry weight and yield, whereas cover crop samples were more closely related to acidity and phenol content. Spontaneous vegetation took an intermediate position, showing a less obvious relationship with either production or compositional characteristics.

A similar tendency was seen in the red cultivars ‘Merlot’ and ‘Pinot Noir’. Bare soil samples were more strongly related to berry weight, yield, pH, and °Brix, whereas cover crop samples were more directly linked to phenolic and anthocyanin variables, as well as titratable acidity. Spontaneous vegetation once again held an intermediate place. These findings point to a consistent distinction between productivity- and maturity-related features under bare soil inter-row management and compositional factors under cover crop management. However, because PCA represents multivariate relationships rather than treatment effects alone, these patterns should not be regarded as proof of direct causality.

The observed connections are consistent with earlier vineyard studies demonstrating that the impacts of groundcover on grape production and composition are substantially influenced by cover crop type, soil, climate, and the intensity and timing of grapevine competition. In a three-year Mediterranean vineyard experiment in Sardinia, Muscas et al. [44] discovered that cover crops lowered grape yield while increasing sugar, anthocyanin, and polyphenol concentrations in comparison to tilled soil. A three-year study in Mediterranean organic vineyards in Chianti indicated that spontaneous vegetation and a legume cover crop might increase yield compared to other groundcover treatments, although the impact on anthocyanins and polyphenols differed depending on management method and year [45].

The full-factorial models accurately explained all grape quality and yield variables, with R^2^ values ranging from 0.799 for °Brix to 0.996 for phenolic compounds. The high explanatory power for phenolic compounds and anthocyanins suggests that the combination of cultivar, management treatment, and year accounted for a significant fraction of the observed variance in these parameters. However, R^2^ values should be considered as indications of model explanatory power, not as evidence that inter-row management alone impacted grape composition.

### 3.5. Implications for Sustainable Vineyard Management and Pollinator Conservation

The findings have a direct impact on developing long-term vineyard management strategies that balance grape quality, productivity, and biodiversity conservation. The association of cover crop management with higher pollinator abundance and grape compositional traits suggests that this strategy may provide diverse ecological services, including pollinator conservation and associated ecosystem services, including soil conservation, and improved wine quality. Similar research has demonstrated that keeping vegetated inter-rows can boost arthropod abundance, and that plant species diversity is a key vegetation trait connected with arthropod populations in Mediterranean vineyards [37].

However, there are challenges to adopting cover crop mixtures in vineyards. Competition for water and nutrients, particularly in areas with water scarcity, is an important barrier to successful implementation [46]. Managing alternating rows with spontaneous vegetation has been suggested as a suitable solution for erosion control, yield support, and grape quality improvement [47]. Research results indicate that spontaneous vegetation treatments may provide intermediate advantages to both pollinators and grape quality, but with greater variability and lower productivity than in cover crop mixtures. Future studies should investigate the long-term viability of these strategies and their interactions with climate variability, soil parameters, and cultivar-specific responses.

### 3.6. Limitations and Opportunities for the Future

Several limitations to this study should be addressed. First, this study was carried out across three growing seasons in a single vineyard; therefore, the applicability of the results to other viticultural locations with various climatic conditions, soil types, and management practices may be limited. Second, while this study focused on the effects of soil inter-row management on vegetation characteristics and pollinator communities, it did not account for the potential influences of the surrounding landscape and semi-natural habitats, or agricultural chemical use, all of which are known to have an impact on pollinator communities in farming environments. Third, the grape quality assessments were confined to basic oenological indicators and did not include sensory evaluations or in-depth wine analyses, which could offer additional information about the impact of management strategies on wine quality. Fourth, the presence of heterogeneity in the model for phenolic compounds, as well as deviations from residual normality in some models, suggests that caution should be applied when interpreting the results, and future research should consider more advanced statistical approaches or larger sample sizes to address these issues.

## 4. Materials and Methods

### 4.1. Study Area and Plant Material

This study was carried out in a commercial vineyard located in the Miniș-Maderat wine-growing area, Arad County, western Romania (46°05′ N, 21°41′ E), during the 2023–2025 growing seasons. The area has a mild continental climate with Mediterranean influences (an average annual temperature of 10–11 °C and annual rainfall of 600–650 mm). The vineyard soils are mainly dark brown forest soils with rendzinas forming on the gently sloping limestone horizon. These soils are rich in stable humic acids, have high cation-exchange capacity and have a neutral to slightly alkaline pH reaction (6.7–7.8).

This study included four wine grape cultivars, two red cultivars, ‘Merlot’ (nine years after planting) and ‘Pinot Noir’ (10 years after planting), and two white cultivars, ‘Sauvignon Blanc’ (13 years) and ‘Italian Riesling’ (10 years). The rows of the vineyard were oriented in a north–south direction, and the grapevines were cultivated under rainfed conditions. All four cultivars were trained on a single Guyot system with a vertical shoot positioning (VSP) trellis (a standard training architecture in temperate European viticulture to balance yield and fruit microclimate). Winter pruning, canopy management during the growing season, protection and fertilisation treatments were carried out according to the same standard commercial practices, uniformly applied in the entire vineyard.

### 4.2. Field Experimental Design

**Figure 7:**
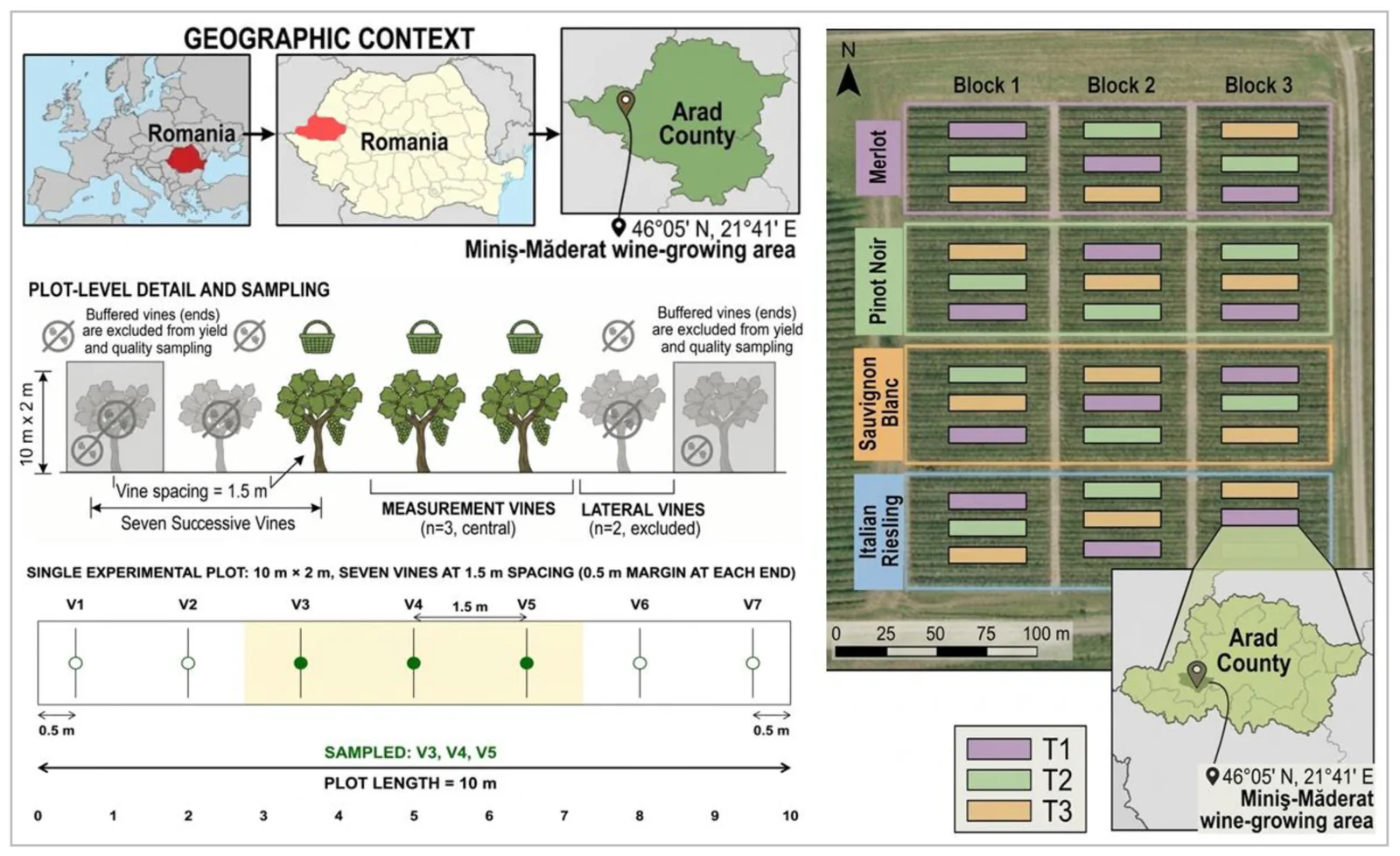
Spatial layout of the 36-plot randomised complete block design (RCBD) across three blocks, four grapevine cultivars (‘Merlot’, ‘Pinot Noir’, ‘Sauvignon Blanc’, and ‘Italian Riesling’), and three inter-row management treatments (T_1_, T_2_, T_3_) in Minis–Maderat vineyard, Arad County, Romania (46°05′ N, 21°41′ E). Plot-level sampling (10 × 2 m) was restricted to the three central measurement vines (V3–V5; green), with four peripheral vines (V1, V2, V6, V7; grey) serving as buffers to prevent edge effects.

The field experiment was designed as a randomised complete block design (RCBD). For each of the four grapevine cultivars (‘Merlot’, ‘Pinot Noir’, ‘Sauvignon Blanc’, and ‘Italian Riesling’), three spatially separated blocks were established along the predominant environmental gradient of the vineyard to account for potential spatial variation in soil characteristics, slope and microclimatic conditions. Within each block, the three inter-row management treatments were randomly assigned to individual plots within the block. Therefore, nine experimental plots (three inter-row management treatments × three blocks) per cultivar were included, generating a total of 36 experimental plots for the four cultivars (Figure 7).

The experimental unit for all statistical analyses was the individual plot (one Cultivar × Block × Treatment combination), monitored over all three years. The 36 plots generated 108 plot-year observations. Quadrat and transect subsamples were aggregated to the plot level before statistical analysis, as described in Section 4.7. The experimental plot size was 10 m × 2 m, with seven successive vines (distance of 1.5 m between vines), corresponding to a planting density of approximately 4444 vines ha^−1^. To minimise potential edge effects, from adjacent treatments, grape yield and berry quality measurements were restricted strictly to the three central vines of each experimental plot. The four peripheral lateral vines (V1, V2 on the left and V6, V7 on the right) served as dedicated spatial buffers and were completely excluded from yield and quality sampling (Figure 7). During the 2023–2025 study period, the same experimental plots and assigned central vines were monitored.

### 4.3. Inter-Row Vegetation Management

Inter-row floor management was established as follows:

(i) Bare soil (BS): Managed by superficial soil tillage (up to 10 cm, three times per year).
(ii) Spontaneous vegetation (SV): Naturally occurring vegetation was maintained throughout the growing season and controlled by mowing to a height of approximately 5 cm.
(iii) Cover crops (CC): A multispecies cover crop mixture was established using the following species, expressed as percentages of seed weight: white clover (*Trifolium repens*, 20%), birdsfoot trefoil (*Lotus corniculatus*, 20%), alfalfa (*Medicago sativa*, 10%), red fescue (*Festuca rubra*, 15%), sainfoin (*Onobrychis viciifolia*, 20%), and yarrow (*Achillea millefolium*, 15%). The total seeding rate was 30 kg ha^−1^**.** The seed mixture was sown in September 2022 and incorporated into the soil at a depth of 2–3 cm across the inter-row area.

Spontaneous vegetation and cover crops were managed by mowing three times a year in late May, mid-July, and early September. Removed vegetation was left in situ as mulch.

#### Vegetation Assessment

Vegetation was sampled twice during May–July, corresponding to the peak flowering period of the component plant species. Five quadrats (1 m × 1 m) were randomly selected in each plot, at least 0.5 m from the edge. Vegetation cover (%), flower density (flowers m^−2^) and plant species richness (number of vascular plant species per quadrat) were noted. Plant diversity was further assessed using the Shannon diversity index (H′), calculated from the relative frequency of occurrence of each plant species within the quadrats. The five quadrat-level observations for each variable (cover, flower density, species richness, and H′) were averaged to obtain a plot-level value for each sampling round. The values from the two sampling rounds were then averaged to obtain an annual value for each plot. All analyses in Section 4.7 used these plot-level annual values. Spontaneous species were identified directly in the field using standard botanical identification criteria; the Pl@ntNet plant identification platform [48], was used as an alternative identification tool for specimens that could not be successfully identified in the field. The sown cover crop mixture was kept throughout the experimental period, and the same management practices were applied for all three years of study. Plant species richness included planted and naturally occurring spontaneous vascular plant species.

### 4.4. Pollinator Sampling

Pollinator communities were studied using fixed transects, established according to the plot dimensions (10 m × 2 m) to ensure spatial alignment between vegetation and pollinator assessments. Observations were conducted annually throughout the season during the peak flowering period (May to July), at two-week intervals. Sampling was limited to days with suitable weather conditions favourable for pollinator activity: air temperature > 18 °C; wind speed < 3 on the Beaufort scale, and no precipitation; surveys were undertaken between 09:00 and 16:00 h.

In each transect, the observer repeatedly walked the entire 10 m transect during a standardised observation period of 15 min and recorded all insects observed visiting or interacting with flowers in the inter-row vegetation. When necessary, individuals were captured using an entomological net for identification and subsequently released. Identification was performed in the field whenever possible and, when necessary, supplemented using the *iNaturalist* identification platform [49] (accessed during the study period). Pollinator abundance was recorded for each transect at each sampling occasion, and observations from the multiple sampling occasions within each year were averaged to calculate the annual mean abundance for each transect.

Pollinator abundance was recorded at each sampling occasion. Repeated observations within each plot and year were aggregated to obtain annual plot-level values for statistical analysis. Pollinators were classified in five groups as follows: honeybees (*Apis mellifera*), wild bees (*Anthophila*; excluding *Apis mellifera* and *Bombus* spp.), bumblebees (*Bombus* spp.), hoverflies (*Syrphidae*), and butterflies (*Lepidoptera*). Annual abundance values were calculated separately for each group and for total pollinator abundance. The repeated transect observations were treated as subsamples within plots rather than as independent experimental replicates.

### 4.5. Grapevine Yield and Berry Quality

Grape yield and berry quality were evaluated at technological maturity during each growing season (2023–2025). The sampling date was adjusted according to the maturity stage of each cultivar. Grapes were collected from the three centrally located vines in each experimental plot to determine grape yield (kg vine^−1^) and berry weight (g).

Total soluble solids (TSS, °Brix) were measured using a digital refractometer (HI96811, Hanna Instruments, Woonsocket, RI, USA). Titratable acidity (TA, g L^−1^ tartaric acid equivalents) was determined by titration with NaOH (Hanna HI84502 mini-titrator; HI84502, Hanna Instruments, Woonsocket, RI, USA), and juice pH was measured using a calibrated pH meter (HI2002, Hanna edge^®^; HI2002, Hanna Instruments, Woonsocket, RI, USA).

For red grape cultivars (Merlot and Pinot Noir), the accumulation of phenolic and anthocyanin compounds was determined in berry skins using spectrophotometric methods. Absorbance measurements were performed using a HI801 Iris visible spectrophotometer (Hanna Instruments, Woonsocket, RI, USA). Total phenolic content was determined using the Folin–Ciocalteu method by measuring absorbance at 765 nm and expressing the results as gallic acid equivalents (mg GAE L^−1^). Total anthocyanin content was determined only in the two red cultivars (Merlot and Pinot Noir) using the pH differential method in buffer solutions at pH 1.0 and 4.5. Absorbance was measured at 520 and 700 nm, and anthocyanin concentration was expressed as cyanidin-3-glucoside equivalents (mg L^−1^). Phenolic content was analysed across all four cultivars, whereas anthocyanin content was analysed only in the two red cultivars.

### 4.6. Pollinator Community Indicators of Habitat Quality

Pollinator community attributes were utilised to assess indirectly habitat quality and ecological conditions in the vineyard agroecosystem. Although grape flowers are self-fertile and primarily wind-pollinated, requiring no insect vectors for fruit set, pollinator abundance and community diversity serve as important indicators of vineyard habitat sustainability. The indicators considered were total pollinator abundance, Shannon diversity (H′), Simpson diversity (1 − D), and the relative composition of the main pollinator groups.

### 4.7. Statistical Analysis

All statistical analyses were performed using R software (version 4.6.1; R Core Team, 2026) and XLSTAT (version 2023.5.1; Addinsoft, Paris, France). Statistical significance was set at *p* < 0.05. Data distributions and model residuals were assessed for normality and homogeneity of variance. When assumptions were not satisfied, data transformations were evaluated, and the model diagnostics were reviewed.

The experimental unit for all analyses was the individual experimental plot (a single Cultivar × Block × Treatment combination; *n* = 36 plots), followed over three years, yielding *n* = 108 plot-year observations. Subsamples obtained at the quadrat level (Section Vegetation Assessment) or transect level (Section 4.4) were averaged to the plot level before modelling to avoid treating subsamples as independent replicates. For all measures of pollinator abundance, repeated observations were aggregated within each plot and year to give one annual value, resulting in a balanced dataset in which each plot was sampled in each year. Therefore, pollinator abundance data were analysed using linear models with treatment, year, and their interaction as fixed effects. This model choice was based on the comparison of model results, rather than on the balanced structure of the dataset alone.

Vegetation characteristics (vegetation cover, flower density, and plant species richness) were analysed using linear mixed-effects models (LMMs) in XLSTAT, with inter-row management treatment, year, and their interaction as fixed effects, and block and experimental plot nested within block as random effects. Significant differences among treatments were assessed using Tukey’s HSD test.

Total pollinator abundance and the abundance of the five pollinator groups (honeybees, wild bees, bumblebees, hoverflies, and butterflies) were analysed using two-way linear models with treatment, year, and their interaction as fixed effects. All repeated transect samples in a plot/year were averaged prior to analyses, so each plot had a single value for each year for each group. The dataset was balanced across treatments and years. Differences among treatments were tested by analysis of variance (ANOVA), with Tukey-adjusted estimated marginal means (EMMs; Figure 3 and Supplementary Table S2). Associations between vegetation parameters and pollinator abundance were assessed using Pearson’s correlation coefficients, followed by linear regression analysis to identify significant associations.

Pollinator community composition was analysed in R using the vegan package. The abundance matrix of the five pollinator groups was square-root transformed and used to calculate Bray–Curtis dissimilarities. Community composition was visualised using two-dimensional NMDS, and differences associated with treatment, year, and their interaction were tested using PERMANOVA with the adonis2 function and 999 permutations. Homogeneity of multivariate dispersion was assessed using PERMDISP (betadisper). When significant treatment effects were detected, pairwise PERMANOVA comparisons were performed using 999 permutations. SIMPER analysis was used to assess the contribution of individual pollinator groups to Bray–Curtis dissimilarities.

Grape yield and berry quality were analysed using full-factorial linear models with cultivar, inter-row vegetation management treatment, year, and their interactions as explanatory factors. Separate models were fitted for soluble solids (°Brix), juice pH, titratable acidity, total phenolic content, total anthocyanin content, berry weight, and grape yield, using the model: “Response variable~Cultivar × Treatment × Year”. Because anthocyanins were measured only in the two red cultivars, a separate model (Anthocyanins~Cultivar × Treatment × Year) was fitted to the red-cultivar subset (*n* = 54 plot-year observations). Treatment means were obtained using estimated marginal means (EMMs) and Tukey-adjusted pairwise comparisons, reported in Table 4 and Table 5.

Model performance was assessed using R^2^ and adjusted R^2^, while residual diagnostics were used to evaluate normality, homogeneity of variance, influential observations, and multicollinearity using the performance package in R.

PCA was used to investigate multivariate relationships among inter-row vegetation management treatments and grape yield and berry quality variables. The general PCA included soluble solids (°Brix), pH, titratable acidity, phenolic content, berry weight, and yield. A separate PCA for Merlot and Pinot Noir additionally included anthocyanin content. PCA biplots were used to visualise variable loadings and relationships among treatments and response variables. The PCA was used as an exploratory and visualisation tool; treatment differences were evaluated using the corresponding statistical models and are reported as estimated means ± SE with Tukey-adjusted *p*-values in Table 4 and Table 5.

## 5. Conclusions

This three-year study identified differences associated with inter-row vegetation management in vineyard floor vegetation, pollinator abundance, and grape quality. Cover crop mixtures had the highest values of vegetation cover, flower density, and plant species richness, followed by spontaneous vegetation and bare soil. Although this ranking was observed across the study period, the magnitude of treatment differences varied among years. Pollinator abundance was positively associated with all three vegetation descriptors, and plant Shannon diversity was the vegetation trait most closely associated with pollinator abundance, both in bivariate correlations and in multiple regression. Inter-row vegetation management explained a substantial proportion of the variation in pollinator community composition.

Grape quality responses differed according to the measured trait and cultivar. Phenolic concentrations were higher under cover crop mixtures than under bare soil in all four cultivars, with larger differences in the red than in the white cultivars. Anthocyanin concentrations were also higher under cover crop mixtures than under bare soil in the two red cultivars. Bare soil was associated with higher yield, berry weight, soluble solids, and pH, whereas cover crop mixtures were associated with higher titratable acidity. Multivariate ordination plots separated treatments along axes associated with productivity-related traits and grape composition. These patterns reflect covariation among measured variables and do not, by themselves, demonstrate a physiological trade-off between yield and grape composition.

Treatment responses were not uniform across all years and cultivars. Significant Treatment × Year interactions were detected for several vegetation variables and for honeybee, bumblebee, and butterfly abundance, while a Treatment × Cultivar interaction was detected for phenolic content. These interactions indicate that the magnitude of treatment-associated differences depended on the response variable and, where applicable, the year or cultivar. Consequently, the results should not be interpreted as evidence of a uniform response across all vineyard conditions.

Overall, the findings show that inter-row vegetation management was associated with quantifiable differences in vegetation resources, pollinator abundance, and grape traits. Spontaneous vegetation and multispecies cover crops were associated with greater vegetation resources and higher pollinator abundance than bare soil, while bare soil was associated with higher yield-related traits and cover crop mixtures with higher phenolic and anthocyanin concentrations. However, the observational nature of the study and the potential influence of other vineyard covariates limit causal interpretation. Inter-row vegetation management may therefore represent a useful component of sustainable vineyard management, but its potential benefits should be evaluated alongside possible competition for water and nutrients, which was not measured in this study. Further multi-year experiments under different environmental conditions, incorporating quantitative assessments of resource competition and evaluations of grape sensory characteristics and resulting wine quality, are needed to determine the reproducibility and practical implications of these findings.

## Figures and Tables

**Table 1:** Effects of year and inter-row management on vegetation characteristics, based on linear mixed-effects models (LMMs).

Response Variable	Source of Variation	df	F-Value	*p*-Value
Vegetation cover (%)	Treatment	2, 16	184.72	<0.001
	Year	2, 16	9.47	0.002
	Treatment × Year	4, 16	3.59	0.027
Flower density (flowers m^−2^)	Treatment	2, 16	267.35	<0.001
	Year	2, 16	12.61	<0.001
	Treatment × Year	4, 16	4.20	0.015
Plant species richness (species m^−2^)	Treatment	2, 16	156.92	<0.001
	Year	2, 16	7.86	0.007
	Treatment × Year	4, 16	2.94	0.045

Treatment and year were set as fixed effects, with block acting as a random effect and the experimental plot acting as the repeated variable. Pairwise comparisons were adjusted using Tukey’s method; *p* < 0.05 was considered statistically significant.

**Table 2:** Multivariate dispersion and PERMANOVA results for pollinator community composition.

Factor	df	PERMDISP (Betadisper *p*)	PERMANOVA R^2^	PERMANOVA *p*
Treatment	2	0.868 ^ns^	0.765	0.001 ***
Year	2	0.982 ^ns^	0.181	0.001 ***
Treatment × Year	4	–	0.025	0.002 **
Residuals	99	–	–	–

PERMANOVA was run on Bray–Curtis dissimilarity matrices (999 permutations). The R^2^ values represent the proportion of variation in pollinator community composition explained by each factor. Homogeneity of multivariate dispersion was assessed using PERMDISP (*betadisper*). ns, not significant (*p* > 0.05); **, *p* < 0.01; ***, *p* < 0.001; df, degrees of freedom. Permutational multivariate analysis of variance (PERMANOVA) results testing the effects of inter-row management treatment (BS, SV, CC), year (2023–2025), and their interaction on pollinator community composition in a commercial vineyard in western Romania.

**Table 3:** Multiple linear regression model explaining variation in total pollinator abundance (*n* = 108).

Parameter	Estimate (B) ± SE	Standardised Coefficient (β)	t-Value	*p*-Value	95% CI
Intercept	−73.734 ± 24.594	—	−2.998	0.0034	−122.498 to −24.969
Flower density (flowers m^−2^)	1.982 ± 0.286	0.491	6.941	<0.0001	1.416 to 2.548
Shannon diversity (H′)	125.592 ± 19.111	0.465	6.572	<0.0001	87.699 to 163.485
Model summary					
Sample size (*n*)	108				
R^2^	0.834				
Adjusted R^2^	0.831				
F_2,105_	263.3 ***				
RMSE	23.932				

B, unstandardised regression coefficient; SE, standard error; β, standardised regression coefficient; t, t-statistic; R^2^, coefficient of determination; RMSE, root mean square error. The model included flower density and plant Shannon diversity as simultaneous predictors of total pollinator abundance. *** *p* < 0.001.

**Table 4:** Treatment means (±SE) and Tukey-adjusted pairwise comparisons for grape yield and quality traits, from full-factorial models (Treatment × Cultivar × Year) (*n* = 108).

Variable	BS	SV	CC	*F* (Treat)	*p*	Tukey Adjusted *p* (BS–CC)	Tukey Adjusted *p* (BS–SV)	Tukey Adjusted *p* (SV–CC)
Yield (kg/vine)	3.14 ± 0.027	2.69 ± 0.027	2.29 ± 0.027	252.88	<0.001	<0.001	<0.001	<0.001
Berry weight (g)	1.45 ± 0.008	1.36 ± 0.008	1.29 ± 0.008	102.72	<0.001	<0.001	<0.001	<0.001
Soluble solids (°Brix)	21.8 ± 0.11	21.3 ± 0.11	20.8 ± 0.11	19.26	<0.001	<0.001	0.0032	0.0177
pH	3.249 ± 0.006	3.216 ± 0.006	3.162 ± 0.006	46.81	<0.001	<0.001	0.0014	<0.001
Titratable acidity (g/L tartaric)	6.85 ± 0.049	7.39 ± 0.049	7.92 ± 0.049	119.29	<0.001	<0.001	<0.001	<0.001
Phenolics (mg GAE/L)	893 ± 6.8	960 ± 6.8	1056 ± 6.8	144.37	<0.001	<0.001	<0.001	<0.001
Anthocyanins (mg/L)	621 ± 7.7	672 ± 7.7	751 ± 7.7	73.08	<0.001	<0.001	<0.001	<0.001

Values are presented as means ± standard errors (SE). *F* (Treat) denotes the F-statistic for the main effect of inter-row vegetation management treatment. Pairwise comparison *p*-values are Tukey-adjusted. Treatment × Year interactions were non-significant for the grape traits (*p* > 0.05). Treatment × Cultivar interactions were non-significant for yield, berry weight, soluble solids (°Brix), pH, and titratable acidity (*p* > 0.05). The Treatment × Cultivar interaction was significant for total phenolic content (*F*_6,72_ = 12.82, *p* < 0.001); therefore, cultivar-specific phenolic comparisons are presented in Table 5. Anthocyanins were measured only in the red cultivars (‘Merlot’ and ‘Pinot Noir’). The Treatment × Cultivar interaction for anthocyanin content was non-significant (*F*_2,36_ = 2.59, *p* = 0.089).

**Table 5:** Phenolics (mg GAE/L) by treatment and cultivar, from the full-factorial model (Treatment × Cultivar × Year).

Cultivar	BS	SV	CC	Tukey *p* BS–SV	Tukey *p* BS–CC	Tukey *p* SV–CC
‘Sauvignon Blanc’	397 ± 13.6	430 ± 13.6	480 ± 13.6	0.201	0.0001	0.030
‘Italian Riesling’	427 ± 13.6	460 ± 13.6	510 ± 13.6	0.201	0.0001	0.030
‘Merlot’	1443 ± 13.6	1545 ± 13.6	1721 ± 13.6	<0.0001	<0.0001	<0.0001
‘Pinot Noir’	1307 ± 13.6	1403 ± 13.6	1513 ± 13.6	<0.0001	<0.0001	<0.0001

Means are marginal means from emmeans (model_phen, ~Treatment|Cultivar), pooled across years. Tukey *p*-values from pairs. The CC > SV > BS ranking held in all four cultivars, but the magnitude of the CC–BS contrast differed strongly between white cultivars (~83 mg GAE/L) and red cultivars (~206–278 mg GAE/L), which is the source of the significant Treatment × Cultivar interaction (*F*_6,72_ = 12.82, *p* < 0.001).

**Table 6:** Variation explained by linear models describing the effects of cultivar, inter-row vegetation management treatment and year on grape yield and quality traits.

Response Variable	R^2^	Adjusted R^2^
Soluble solids (°Brix)	0.799	0.701
pH	0.937	0.907
Titratable acidity	0.944	0.917
Phenolic compounds	0.996	0.994
Anthocyanins	0.980	0.971
Berry weight	0.915	0.873
Yield	0.946	0.920

R^2^ is the proportion of variance explained by the linear model, while adjusted R^2^ reflects the number of predictors in the model.

## Data Availability

Data are contained within the article and Supplementary Materials.

## Supplementary Materials

The following supporting information can be downloaded at: https://www.mdpi.com/article/10.3390/plants15213405/s1, Table S1: Treatment means (±SE) and Tukey-adjusted pairwise *p*-values for vegetation variables by year, from linear models with Treatment, Year and Block as fixed effects (*n* = 108 plot-year observations)/Means are estimated marginal means from the model. Tukey *p*-values are from pairs (emmeans(…), adjust = Tukey). BS = bare soil, SV = spontaneous vegetation, CC = cover-crop mixture. Species richness was analysed separately (Figure 1C); treatment × year comparisons for this trait are presented graphically rather than tabulated. Table S2: Annual treatment means (± SE) and Tukey-adjusted pairwise *p*-values for pollinator groups under different inter-row vegetation management treatments (2023–2025)/Values are estimated marginal means (±SE) from the fitted models. Pairwise *p*-values are adjusted using Tukey’s method. BS = bare soil; SV = spontaneous vegetation; CC = cover-crop mixture. Pollinator groups comprise honeybees, solitary (wild) bees, hoverflies, bumblebees, and butterflies. The analysis includes 36 experimental plots monitored over three years (2023–2025), yielding 108 plot-year observations.

## Abbreviations

The following abbreviations are used in this manuscript:

Inter-row vegetation management treatment	
**BS**	Bare soil
**SV**	Spontaneous vegetation
**CC**	Cover crops/cover crop mixture
*Statistics and analysis methodology*	
**ANOVA**	Analysis of Variance
**β (Beta)**	Standardised regression coefficient
**CI**	Confidence interval
**df**	Degrees of freedom
**EMMs**	Estimated marginal means
**F**	F-statistic/F-value
**H′**	Shannon diversity index
**HSD**	Honest significant difference (Tukey HSD)
**LMMs/LMM**	Linear mixed-effects models
**NMDS**	Non-metric multidimensional scaling
**ns**	Not significant
***p***	*p*-value
**PCA**	Principal component analysis
**PC1/PC2**	Principal component 1/2
**PERMANOVA**	Permutational multivariate analysis of variance
**PERMDISP**	Permutational analysis of multivariate dispersions
**r**	Pearson correlation coefficient
**R^2^**	Coefficient of determination
**RCBD**	Randomised complete block design
**RMSE**	Root mean square error
**SD**	Standard deviation
**SE**	Standard error
**SIMPER**	Similarity percentages
**t**	t-statistic/t-value
**VIF**	Variance inflation factor
*Physicochemical and quality parameters of grapes*	
**GAE**	Gallic acid equivalents
**NaOH**	Sodium hydroxide
**pH**	Potential of hydrogen
**TA**	Titratable acidity
**TSS**	Total soluble solids
