# Supplementary material for: Vineyard Floor Management as a Multifunctional Strategy for Pollinator Communities and Grape Quality
Source: Plants (Basel). 2026 Nov 2;15(21):3405. doi: 10.3390/plants15213405 (PMC13765702; doi:10.3390/plants15213405)
Supplement: Supplementary file 1 [file plants-15-03405-s001.zip › plants-4624749-supplementary.pdf]

Supplementary Table S1. Treatment means (± SE) and Tukey-adjusted pairwise p-values for vegetation variables by year, from linear models with Treatment, Year and Block as fixed effects (n = 108 plot-year observations); (2.1. Effects of inter-row management on vineyard-floor vegetation)

Variable	Year	BS	SV	CC	Tukey–adjusted p (BS–SV)	Tukey–adjusted p (BS–CC)	Tukey–adjusted p (SV–CC)
Vegetation cover (%)	2023	19.8 ± 1.41	71.1 ± 1.41	86.8 ± 1.41	<0.0001	<0.0001	<0.0001
	2024	21.2 ± 1.41	73.9 ± 1.41	84.6 ± 1.41	<0.0001	<0.0001	<0.0001
	2025	21.4 ± 1.41	73.8 ± 1.41	87.8 ± 1.41	<0.0001	<0.0001	<0.0001
Flower density (flowers m^{-2})	2023	3.72 ± 1.27	28.68 ± 1.27	36.55 ± 1.27	<0.0001	<0.0001	<0.0001
	2024	4.67 ± 1.27	29.20 ± 1.27	34.22 ± 1.27	<0.0001	<0.0001	0.0171
	2025	4.49 ± 1.27	30.26 ± 1.27	39.17 ± 1.27	<0.0001	<0.0001	<0.0001
Shannon diversity (H′)	2023	1.29 ± 0.031	1.58 ± 0.031	1.73 ± 0.031	<0.0001	<0.0001	0.0020
	2024	1.32 ± 0.031	1.58 ± 0.031	1.80 ± 0.031	<0.0001	<0.0001	<0.0001
	2025	1.32 ± 0.031	1.58 ± 0.031	1.80 ± 0.031	<0.0001	<0.0001	<0.0001

Means are estimated marginal means from the model. Tukey p-values are from pairs (emmeans(...), adjust = Tukey). BS = bare soil, SV = spontaneous vegetation, CC = cover-crop mixture. Species richness was analysed separately (Figure 1C); treatment × year comparisons for this trait are presented graphically rather than tabulated.

Supplementary Table S2. Annual treatment means (± SE) and Tukey-adjusted pairwise *p*-values for pollinator groups under different inter-row vegetation management treatments (2023–2025). (2.3. Responses of pollinator groups to inter-row vegetation management)

Group	Year	BS	SV	CC	Tukey–adjusted *p* (BS–SV)	Tukey–adjusted *p* (BS–CC)	Tukey–adjusted *p* (SV–CC)
Honeybees	2023	25.8 ± 1.43	30.7 ± 1.43	39.6 ± 1.43	0.064	<0.001	0.0010
	2024	27.6 ± 1.43	34.5 ± 1.43	39.7 ± 1.43	0.0084	<0.001	0.0459
	2025	30.1 ± 1.43	36.0 ± 1.43	46.2 ± 1.43	0.0234	<0.001	0.0002
Solitary (wild) bees	2023	17.7 ± 1.01	28.3 ± 1.01	35.7 ± 1.01	<0.0001	<0.0001	0.0002
	2024	22.0 ± 1.01	34.3 ± 1.01	45.7 ± 1.01	<0.0001	<0.0001	<0.0001
	2025	25.3 ± 1.01	41.7 ± 1.01	55.7 ± 1.01	<0.0001	<0.0001	<0.0001
Hoverflies	2023	9.0 ± 0.76	15.0 ± 0.76	20.3 ± 0.76	<0.0001	<0.0001	0.0003
	2024	12.0 ± 0.76	19.3 ± 0.76	27.3 ± 0.76	<0.0001	<0.0001	<0.0001
	2025	15.0 ± 0.76	24.3 ± 0.76	34.3 ± 0.76	<0.0001	<0.0001	<0.0001
Bumblebees	2023	12.3 ± 1.16	24.3 ± 1.16	31.7 ± 1.16	<0.0001	<0.0001	0.0008
	2024	16.3 ± 1.16	30.7 ± 1.16	41.0 ± 1.16	<0.0001	<0.0001	<0.0001
	2025	20.3 ± 1.16	37.7 ± 1.16	51.0 ± 1.16	<0.0001	<0.0001	<0.0001
Butterflies	2023	4.33 ± 0.57	9.00 ± 0.57	12.00 ± 0.57	<0.0001	<0.0001	0.0040
	2024	5.33 ± 0.57	12.00 ± 0.57	15.67 ± 0.57	<0.0001	<0.0001	0.0007
	2025	6.33 ± 0.57	15.00 ± 0.57	20.33 ± 0.57	<0.0001	<0.0001	<0.0001

Values are estimated marginal means (± SE) from the fitted models. Pairwise *p*-values are adjusted using Tukey's method. BS = bare soil; SV = spontaneous vegetation; CC = cover-crop mixture. Pollinator groups comprise honeybees, solitary (wild) bees, hoverflies, bumblebees, and butterflies. The analysis includes 36 experimental plots monitored over three years (2023–2025), yielding 108 plot-year observations.
